# The thrombotic burden of high-risk immune thrombocytopenia patients receiving second-line treatments: a systematic review

**DOI:** 10.1093/rheumatology/keag461

**Published:** 2026-08-26

**Authors:** Elisa Tarasconi, Bianca Clerici, Giulia Marcucci, Eleonora Pontisso, Chiara Mondini, Chiara Pisetta, Simone Birocchi, Tommaso Schioppo, Gian Marco Podda

**Affiliations:** Dipartimento di Scienze della Salute (DiSS), University of Milan, S.C. Medicina Generale SP, Hospital San Paolo, ASST Santi Paolo e Carlo, Milan, Italy; Dipartimento di Scienze della Salute (DiSS), University of Milan, S.C. Medicina Generale SP, Hospital San Paolo, ASST Santi Paolo e Carlo, Milan, Italy; Dipartimento di Scienze della Salute (DiSS), University of Milan, S.C. Medicina Generale SP, Hospital San Paolo, ASST Santi Paolo e Carlo, Milan, Italy; Dipartimento di Scienze della Salute (DiSS), University of Milan, S.C. Medicina Generale SP, Hospital San Paolo, ASST Santi Paolo e Carlo, Milan, Italy; Dipartimento di Scienze della Salute (DiSS), University of Milan, S.C. Medicina Generale SP, Hospital San Paolo, ASST Santi Paolo e Carlo, Milan, Italy; Dipartimento di Scienze della Salute (DiSS), University of Milan, S.C. Medicina Generale SP, Hospital San Paolo, ASST Santi Paolo e Carlo, Milan, Italy; Dipartimento di Scienze della Salute (DiSS), University of Milan, S.C. Medicina Generale SP, Hospital San Paolo, ASST Santi Paolo e Carlo, Milan, Italy; Dipartimento di Scienze della Salute (DiSS), University of Milan, S.C. Medicina Generale SP, Hospital San Paolo, ASST Santi Paolo e Carlo, Milan, Italy; Dipartimento di Scienze della Salute (DiSS), University of Milan, S.C. Medicina Generale SP, Hospital San Paolo, ASST Santi Paolo e Carlo, Milan, Italy

**Keywords:** purpura, thrombocytopenic, idiopathic, autoimmunity, antiphospholipid syndrome, lupus erythematosus, systemic, thrombosis, receptors, thrombopoietin

## Abstract

**Objective:**

Immune thrombocytopenia (ITP) carries an increased thrombotic risk, especially when associated with autoimmune conditions and/or antiphospholipid antibody (aPL) positivity. The burden of thrombotic events in patients treated with second-line therapies in these instances has not been systematically studied.

**Methods:**

Medline and Embase were searched from 2010 to 16 November 2025, for studies on high-risk ITP patients receiving eligible second-line treatments, including thrombopoietin receptor agonists (TPO-RAs), fostamatinib, rituximab, other immune suppressants (ciclosporin, cyclophosphamide, azathioprine and mycophenolate) and splenectomy. High-risk ITP was defined as (i) primary ITP (pITP) with aPL positivity or (ii) secondary ITP (sITP) when associated to an autoimmune disease posing an increased thrombotic risk. The primary outcome was the frequency of any thrombotic event during follow-up. Secondary outcomes were the separate frequencies of arterial and venous thromboses, overall and stratified by treatment.

**Results:**

We included 13 studies providing data of 308 high-risk ITP patients on at least one eligible second-line treatment. Thrombosis occurred in 40 patients (10%, 95% CI 5–19) over a median follow-up ranging from 6 to 42 months. Stratifying by treatment, thrombosis occurred in 30 patients on TPO-RAs (16%, 95% CI 11–22), in 3 on rituximab (14%, 95% CI 3–35), in 3 on immune suppressants (11%, 95% CI 2–28) and in 6 who underwent splenectomy (12%, 95% CI 4–24).

**Conclusion:**

We have found a consistent thrombotic burden in high-risk ITP patients on second-line therapy, evident across all examined treatment options, underscoring the pro-thrombotic immune *milieu* of this population and the need for tailored risk-mitigation strategies.

Rheumatology key messages

- High-risk ITP patients receiving second-line treatments showed a clinically relevant thrombotic burden.
- Available evidence does not permit treatment-specific safety comparisons.
- aPL positivity and autoimmune *milieu* may be key determinants of thrombosis.

## Background

Immune thrombocytopenia (ITP) is an acquired immune-mediated disease characterized by a transient or persistent decrease in the platelet count. Primary ITP (pITP) is defined by isolated thrombocytopenia without any identifiable underlying cause or associated disorder, whereas secondary ITP (sITP) occurs in association with another condition, typically of immune or infectious origin [1, 2]. Due to their low platelet counts, ITP patients are at increased risk of bleeding, but they also exhibit an increased risk of both arterial and venous thrombosis compared with the disease-free population [3]. The thrombotic risk is believed to be greater in certain subgroups of ITP patients, such as those with pITP and antiphospholipid antibody (aPL) positivity [4] and those with ITP secondary to immune-mediated conditions that further increase the thrombotic risk, such as systemic lupus erythematosus (SLE), antiphospholipid antibody syndrome (APS), Evans’ syndrome, inflammatory bowel diseases (IBD) or rheumatoid arthritis (RA) [5–13]. Patients with ITP may require treatment to raise their platelet count to safe levels. The first-line treatment for ITP consists of corticosteroids and/or intravenous immunoglobulin (IVIG), the latter being reserved to specific clinical settings such as severe or life-threatening bleeding, pregnancy and children. For patients who are refractory to or relapse after first-line therapy, available second-line treatments include thrombopoietin receptor agonists (TPO-RAs), fostamatinib, rituximab, immune suppressants and splenectomy [14]. While all are effective in raising platelet counts, they have been associated with side effects, with thrombosis being a significant safety concern. Several studies have reported arterial and venous thrombotic events in patients receiving TPO-RAs [15–17], although most comparative studies have not found a statistically significant difference in the rates of thromboembolic events of TPO-RAs users and non-users [18, 19]. Fostamatinib, a recently introduced oral Syk inhibitor, appears to carry a lower thrombotic risk than TPO-RAs [20, 21]. Similarly, rituximab and immune suppressants do not seem to be associated with an increase in thrombosis rates [22–24]. On the contrary, thrombosis, both arterial and venous, is a well-known potential complication of splenectomy [25, 26]. However, the burden of thrombosis in ITP patients at high thrombotic risk due to immunological mechanisms treated with second-line therapies has not been systematically studied. To this end, we have conducted a systematic review of published studies.

## Methods

### Data sources and search strategy

Medline and Embase were searched independently by two reviewers (G.M. and E.T.) from 2010 to 16 November 2025, using a predefined research string (Supplementary Data S1) and a snowball strategy.

### Study selection, eligibility criteria and definitions

Eligibility assessment was performed independently by two reviewers (G.M. and E.T.). Disagreements were resolved by consensus or with the intervention of a third reviewer (B.C.). We included peer-reviewed studies in English with available data on thrombotic events in patients with primary or secondary ITP at high thrombotic risk due to immunological mechanisms on treatment with at least one eligible second-line treatment. Eligible second-line treatments included TPO-RAs, fostamatinib, rituximab, other immune suppressants (ciclosporin, cyclophosphamide, azathioprine and mycophenolate) and splenectomy. Our definition of high-risk ITP encompassed two categories: (1) pITP with positive aPL not meeting clinical criteria for APS; and (2) sITP in the setting of immune-mediated conditions associated with an increased thrombotic risk based on existing literature [5–13], including SLE, APS, Evans’ syndrome, IBD and RA. aPL positivity was defined as the positivity of any tested aPL, as reported by the authors. We excluded studies describing fewer than 5 patients and congress abstracts.

### Study outcomes

The primary outcome was the frequency of thrombotic events in patients with high-risk ITP. Secondary outcomes included the frequencies of venous and arterial thrombotic events in high-risk ITP patients. Thrombosis was defined as any fatal or non-fatal thrombotic event involving the arterial and/or venous district.

### Data extraction

Data extraction was performed independently by two reviewers (G.M. and E.T.) and cross-checked for correctness and consistency by a third investigator (B.C.), using a pre-defined electronic case report form (eCRF) set up on Microsoft Excel. We collected the following data, when available: eligible patients’ sex, age, ITP diagnosis (primary *vs* secondary), aPL profile (positive *vs* negative), conditions associated with ITP (SLE, APS, Evans’ syndrome, IBD and/or RA), administered eligible second-line treatment(s), number of previous lines of therapy, follow-up duration, primary and secondary outcome events. We performed conversions of the length of follow-up duration in months, where necessary.

### Statistical analysis

We presented data as medians with interquartile ranges (IQR) or proportions and counts, as appropriate. We calculated the unadjusted frequencies of thrombosis (any thrombosis, arterial and venous) for the whole cohort (high-risk primary and secondary ITP patients). We calculated 95% confidence intervals (CI) with the exact binomial method (GraphPad Prism). We performed *post-hoc* subgroup analyses stratifying patients by disease group (pITP, sITP, SLE, SLE with positive aPL not meeting criteria for APS, APS, Evans’ syndrome) and by administered second-line treatment(s) (TPO-RAs, rituximab, other immune suppressants, splenectomy). We performed meta-analysis of the data of the whole cohort and of the most numerically consistent disease subgroups using the random intercept logistic regression model and logit transformation. Heterogeneity was measured using the *I*^2^ statistic and the Wald test (R Core Team software, 2023). The unadjusted frequencies of patients with arterial and venous thrombosis were compared using the *χ*^2^ test. Two-tailed *P*-values below 0.05 were considered statistically significant. The meta-analysis of frequencies was performed using R version 4.4.5 with the meta package. No imputation of missing data was performed. Risk of bias assessment of all eligible studies was performed independently by E.T. and B.C. using the ROBINS-I V2 tool. The protocol of this systematic review was not registered. The results are reported in accordance with the PRISMA checklist (*attached*). The raw data supporting the results of this study will be made available upon reasonable request.

## Results

### Description of eligible studies

We retrieved 5876 citations through our literature search. After the exclusion of duplicates (*n* = 468) and ineligible and unretrievable studies (*n* = 5395), we included 13 studies [27–39] providing data on the primary outcome for a total of 308 high-risk ITP patients on at least one eligible second-line treatment (Supplementary Fig. S1). Based on the available data (Supplementary Table S1), most patients were female (156/208, 75%). The median age, among studies reporting this information, ranged from a minimum of 32 to a maximum of 61 years. Fourteen patients had high-risk pITP and the remaining 294 had high-risk sITP. SLE was the most prevalent cause of sITP (*n* = 232), followed by Evans’ syndrome (*n* = 34), APS (*n* = 29) and RA (*n* = 2). Ten patients had both SLE and APS. We retrieved no eligible studies describing patients with ITP secondary to IBD. Among high-risk ITP patients with known outcome by treatment, 188 patients received TPO-RAs, 22 were treated with rituximab, 28 with immune suppressants and 51 underwent splenectomy. None of the eligible patients received fostamatinib. A combination therapy with another eligible second-line treatment was reported for 8 of the patients treated with rituximab and for 27 patients on immune suppressants. Median follow-up duration, reported in less than half of the eligible studies, ranged from a minimum of 6 months to a maximum of 42 months.

### Risk of bias assessment

In accordance with the ROBINS-I V2 tool, we considered all eligible studies at critical risk of bias, as none have made attempts at controlling confounding with regards to treatment selection.

### Pooled estimates of thrombotic events in patients with high-risk ITP

A thrombotic event was reported in 40 high-risk ITP patients (meta-analysed frequency: 10%, 95% CI 5–19; *I*^2^ = 0%, *P* = 0.74 for heterogeneity) over a median follow-up—based on available data—ranging from 6 to 42 months (Table 1). Arterial thrombosis occurred in 20 patients (where specified: seven myocardial infarctions, one atrial thrombosis, two retinal thromboses, five strokes, four cases of catastrophic APS, one splenic infarction, one superior mesenteric artery occlusion and three arterial thromboses at unknown location), while 16 patients experienced a venous event (where specified: nine pulmonary embolisms, one splanchnic vein thrombosis, four cerebral venous sinus thromboses, five deep vein thromboses and two thrombophlebitises) (meta-analysed frequencies: 3%, 95% CI 1–13 *vs* 6%, 95% CI 3–10, respectively). For six patients from two eligible studies, the type of thrombosis (arterial *vs* venous) was not specified. We found no significant difference between the frequencies of high-risk ITP patients with arterial and venous thrombosis (*P* = 0.49).

**Table 1:** Primary and secondary outcome events in high-risk ITP patients.

Study	Patients with events/total patients	% (95% CI)	Between-study heterogeneity, *I*^2^; *P*
Thrombosis			
Li *et al*. [27]	0/11	0 (0–28)	
Chen *et al*. [28]	1/10	0 (0–45)	
Gudbrandsdottir *et al*. [29]	0/2	0 (0–84)	
Zhou *et al*. [30]	0/20	0 (0–17)	
Guitton *et al*. [31]	5/18	28 (10–53)	
Frison *et al*. [32]	4/16	25 (7–52)	
Shobha *et al*. [33]	0/12	0 (0–26)	
Gonzalez-Lopez *et al*. [34]	1/14	7 (0–34)	
Fattizzo *et al*. [35]	5/29	17 (6–36)	
Roussotte *et al*. [36]	7/84	8 (3–16)	
Li *et al*. [37]	0/7	0 (0–41)	
Jiang *et al*. [38]	0/5	0 (0–52)	
Marques *et al*. [39]	17/80	21 (13–32)	
Crude proportion	40/308	NA	NA
**Random-effects pooled estimate**	NA	**10 (5–19)**	**0%; 0.74**
Arterial thrombosis			
Li *et al*. [27]	0/11	0 (0–29)	
Chen *et al*. [28]	0/10	0 (0–31)	
Gudbrandsdottir *et al*. [29]	0/2	0 (0–84)	
Zhou *et al*. [30]	0/20	0 (0–17)	
Guitton *et al*. [31]	4/18	22 (6–48)	
Frison *et al*. [32]	1/16	6 (0–30)	
Shobha *et al*. [33]	0/12	0 (0–26)	
Gonzalez-Lopez *et al*. [34]	0/14	0 (0–23)	
Roussotte *et al*. [36]	3/84	4 (1–10)	
Li *et al*. [37]	0/7	0 (0–41)	
Jiang *et al*. [38]	0/5	0 (0–52)	
Marques *et al*. [39]	12/80	15 (8–25)	
Crude proportion	20/279	NA	NA
**Random-effects pooled estimate**	NA	**3 (1–13)**	**0%; 0.73**
Venous thrombosis			
Li *et al*. [27]	0/11	0 (0–29)	
Chen *et al*. [28]	1/10	10 (0–45)	
Gudbrandsdottir *et al*. [29]	0/2	0 (0–84)	
Zhou *et al*. [30]	0/20	0 (0–17)	
Guitton *et al*. [31]	1/18	6 (0–27)	
Frison *et al*. [32]	3/16	19 (4–46)	
Shobha *et al*. [33]	0/12	0 (0–27)	
Gonzalez-Lopez *et al*. [34]	1/14	7 (0–34)	
Roussotte *et al*. [36]	3/84	4 (1–10)	
Li *et al*. [37]	0/7	0 (0–41)	
Jiang *et al*. [38]	0/5	0 (0–52)	
Marques *et al*. [39]	7/80	9 (4–17)	
Crude proportion	16/279	NA	NA
**Random-effects pooled estimate**	NA	**6 (3–10)**	**0%; 0.95**

CI, confidence interval; NA, not applicable.

Crude totals/proportions are reported descriptively and may differ from random-effects pooled estimates, reported in bold in the row ‘Random-effects pooled estimate’ and corresponding to pooled meta-analytic estimates.

The median follow-up—based on data available for less than half of eligible studies—ranged from 6 to 42 months.

The breakdown of patients with arterial and venous thrombosis does not sum up to the total of patients with thrombosis because the type of thrombosis (arterial *vs* venous) was not specified for six patients from two studies.

The difference in the frequencies of patients with arterial and venous thrombosis was not statistically significant (*P* = 0.49).

Between-study heterogeneity was assessed using the *I*² statistic. The *P*-value refers to heterogeneity (Wald test for the random-effect variance in the random-intercept logistic model).

### Pooled estimates of thrombotic events in relevant disease subgroups

We derived data on pITP patients from only one of the eligible studies [32], which reported on 14 subjects with pITP and aPL positivity. Seven (50%) had a triple positive aPL profile. Thrombotic events were observed in three patients (21%, 95% CI 5–51), all involving the venous system (Supplementary Table S2). The length of follow-up was unavailable. Among the patients with high-risk sITP (*n* = 294), 37 experienced a thrombotic event (meta-analysed frequency: 9%, 95% CI 5–19; *I*^2^ = 0%, *P* = 0.69 for heterogeneity) during a median follow-up ranging–based on available data–from 6 to 42 months, with 20 cases of arterial thrombosis, 13 of venous thrombosis and 6 thromboses of unknown type (Supplementary Table S3). We performed *post-hoc* subgroup analyses of the most numerically consistent disease subgroups. The meta-analysed frequencies of thrombosis were 8% (95% CI 4–16; *I*^2^ = 0%, *P* = 0.98 for heterogeneity; median follow-up ranging from 6 to 42 months) in the ‘SLE’ subgroup, 26% (95% CI 95% CI 14–41, *P* = 0.96 for heterogeneity; median follow-up ranging from 6 to 15 months) in the ‘SLE associated with aPL positivity not meeting criteria for APS’ subgroup and 45% (95% CI 28–63; *I*^2^ = 0%, *P* = 0.60 for heterogeneity; median follow-up of 15 months, available for only one eligible study) in the ‘APS’ subgroup (Tables 2 and 3, Supplementary Table S4). We found no statistically significant difference between the proportion of pAPS and sAPS patients with thrombotic events (2/7 *vs* 3/6; *P= 0*.59) (Supplementary Table S5).

**Table 2:** Subgroup analysis by disease group: primary and secondary outcome events of patients with ITP secondary to systemic lupus erythematosus (SLE).

Study	Patients with events/total patients	% (95% CI)	Between-study heterogeneity, *I*^2^; *P*
Thrombosis			
Li *et al*. [27]	0/11	0 (0–29)	
Chen *et al*. [28]	1/10	10 (0–45)	
Gudbrandsdottir *et al*. [29]	0/1	0 (0–98)	
Zhou *et al*. [30]	0/20	0 (0–17)	
Guitton *et al*. [31]	2/13	15 (2–45)	
Frison *et al*. [32]	1/2	50 (1–99)	
Shobha *et al*. [33]	0/12	0 (0–27)	
Gonzalez-Lopez *et al*. [34]	0/3	0 (0–71)	
Roussotte *et al*. [36]	7/84	8 (3–16)	
Li *et al*. [37]	0/7	0 (0–41)	
Jiang *et al*. [38]	0/5	0 (0–52)	
Marques *et al*. [39]	9/64	14 (7–25)	
Crude proportion	20/232	NA	NA
**Random-effects pooled estimate**	NA	**8 (4–16)**	**0%; 0.98**
Arterial thrombosis			
Li *et al*. [27]	0/11	0 (0–29)	
Chen *et al*. [28]	0/10	0 (0–31)	
Gudbrandsdottir *et al*. [29]	0/1	0 (0–98)	
Zhou *et al*. [30]	0/20	0 (0–17)	
Guitton *et al*. [31]	1/13	8 (0–36)	
Frison *et al*. [32]	1/2	50 (1–99)	
Shobha *et al*. [33]	0/12	0 (0–27)	
Gonzalez-Lopez *et al*. [34]	0/3	0 (0–71)	
Roussotte *et al*. [36]	3/84	4 (1–10)	
Li *et al*. [37]	0/7	0 (0–41)	
Jiang *et al*. [38]	0/5	0 (0–52)	
Marques *et al*. [39]	5/64	8 (3–17)	
Crude proportion	10/232	NA	NA
**Random-effects pooled estimate**	NA	**4 (2–8)**	**0%; 0.94**
Venous thrombosis			
Li *et al*. [27]	0/11	0 (0–29)	
Chen *et al*. [28]	1/10	10 (0–45)	
Gudbrandsdottir *et al*. [29]	0/1	0 (0–98)	
Zhou *et al*. [30]	0/20	0 (0–17)	
Guitton *et al*. [31]	1/13	8 (0–36)	
Frison *et al*. [32]	0/2	0 (0–84)	
Shobha *et al*. [33]	0/12	0 (0–27)	
Gonzalez-Lopez *et al*. [34]	0/3	0 (0–71)	
Roussotte *et al*. [36]	3/84	4 (1–10)	
Li *et al*. [37]	0/7	0 (0–41)	
Jiang *et al*. [38]	0/5	0 (0–52)	
Marques *et al*. [39]	6/64	9 (4–19)	
Crude proportion	11/232	NA	NA
**Random-effects pooled estimate**	NA	**5 (2–10)**	**0%; 1**

CI, confidence interval; NA, not applicable.

Crude totals/proportions are reported descriptively and may differ from random-effects pooled estimates.

The median follow-up—based on available data—ranged from 6 to 42 months.

The difference in the frequencies of patients with arterial and venous thrombosis was not statistically significant (*P* = 0.82).

Between-study heterogeneity was assessed using the *I*² statistic. The *P*-value refers to heterogeneity (Wald test for the random-effect variance in the random-intercept logistic model).

**Table 3:** Subgroup analysis by disease group: primary and secondary outcome events of patients with ITP secondary to antiphospholipid antibody syndrome (APS).

Study	Patients with events/Total patients	% (95% CI)	Between-study heterogeneity, I^2^; p
Thrombosis			
Guitton *et al*. [31]	3/5	60 (15–95)	
Gonzalez-Lopez *et al*. [34]	1/5	20 (1–72)	
Fattizzo *et al*. [35]	1/3	33 (1–91)	
Marques *et al*. [39]	8/16	50 (25–75)	
Crude proportion	13/29	NA	NA
**Random-effects pooled estimate**	NA	**45 (28–63)**	**0%; 0.60**

CI, confidence interval; NA, not applicable.

Crude totals/proportions are reported descriptively and may differ from random-effects pooled estimates.

The median follow-up was reported in only one of the eligible studies (15 months).

Patients with both primary and secondary antiphospholipid antibody syndrome were included in this analysis.

Between-study heterogeneity was assessed using the *I*² statistic. The *P*-value refers to heterogeneity (Wald test for the random-effect variance in the random-intercept logistic model).

### Subgroup analyses by administered treatment(s)

Stratifying high-risk ITP patients by administered second-line treatment(s), thrombosis occurred in 30 patients on TPO-RAs (16%, 95% CI 11–22), in 3 patients treated with rituximab (14%, 95% CI 3–35), in 3 patients on immune suppressants (11%, 95% CI 2–28) and in 6 of those who underwent splenectomy (12%, 95% CI 4–24) (Table 4). Data were overall consistent when we stratified patients with SLE, who made up most of the study cohort, based on administered second-line treatment(s) (Table 5). The rates of thrombosis ranged from 10% (95% CI 5–17) in patients on TPO-RAs to 17% (95% CI 2–48) in patients treated with rituximab. All SLE patients on immune suppressants other than rituximab were on combination treatment with another eligible second-line treatment.

**Table 4:** Subgroup analysis by treatment: primary and secondary outcome events of high-risk ITP patients stratified by eligible second-line treatment.

	Patients with thrombotic events	Patients with arterial thrombosis	Patients with venous thrombosis
	Crude proportion, *n*/*N* (%, 95% CI)	Crude proportion, *n*/*N* (%, 95% CI)	Crude proportion, *n*/*N* (%, 95% CI)
All patients	40/308 (13, 9–17)	20/279 (7, 4–11)	16/279 (6, 3–9)
Patients stratified by treatment			
TPO-RAs^a^	30/188 (16, 11–22)	16/143 (11, 7–18)	10/143 (7, 3–13)
Rituximab^b^	3/22 (14, 3–35)	1/22 (5, 0–23)	2/22 (9, 1–29)
Immune suppressants^c^	3/28 (11, 2–28)	1/28 (4, 0–18)	2/28 (7, 1–24)
Splenectomy	6/51 (12, 4–24)	3/51 (6, 1–16)	3/51 (6, 1–16)

CI, confidence interval; TPO-RAs, thrombopoietin receptor agonists.

All estimates in this table are crude proportions based on aggregated counts; no random-effects pooling was performed.

Median follow-up range: 6–42 months.

The breakdown of arterial and venous thrombotic events was unavailable for five eligible patients with thrombotic events from the same study. Among the 279 patients with available breakdown of arterial *vs* venous thrombosis, the type of thrombosis was not specified in 1 patient.

The total of thrombotic events stratified by eligible second-line therapy exceeds the number of total thrombotic events because of combination therapies.

a Avatrombopag (*n* = 1), eltrombopag (*n* = 149), romiplostim (*n* = 66).

b In combination with another second-line treatment (*n* = 8).

c In combination with other eligible second-line treatments (*n* = 27).

**Table 5:** Subgroup analysis by disease group and treatment: primary and secondary outcome events of patients with ITP secondary to systemic lupus erythematosus stratified by eligible second-line treatment.

	Patients with thrombotic events	Patients with arterial thrombosis	Patients with venous thrombosis
	Crude proportion, *n*/*N* (%, 95% CI)	Crude proportion, *n*/*N* (%, 95% CI)	Crude proportion, *n*/*N* (%, 95% CI)
All patients^a^	20/232 (9, 5–13)	10/232 (4, 2–8)	11/232 (5, 2–8)
Patients stratified by treatment			
TPO-Ras	12/121 (10, 5–17)	6/105 (6, 2–12)	7/105 (7, 3–13)
Rituximab^b^	2/12 (17, 2–48)	1/12 (8, 0–39)	1/12 (8, 0–39)
Immune suppressants^c^	1/19 (5, 0–26)	1/19 (5, 0–26)	0/19 (0, 0–18)
Splenectomy	6/50 (12, 5–24)	3/50 (6, 1–17)	3/50 (6, 1–17)

CI, confidence interval; TPO-RAs, thrombopoietin receptor agonists.

All estimates in this table are crude proportions based on aggregated counts; no random-effects pooling was performed.

Median follow-up range: 6–42 months.

Among patients with a thrombotic event, the type of thrombosis (arterial *vs* venous) was unavailable for one patient. The breakdown of patients with arterial and venous thrombotic events does not sum up to the total number of patients with thrombotic events because two patients had both arterial and venous thrombosis.

The total of thrombotic events stratified by eligible second-line therapy exceeds the number of total thrombotic events because of combination therapies.

a Concomitant antiphospholipid antibody syndrome (*n* = 25), concomitant Evans’ syndrome (*n* = 33), positive antiphospholipid antibodies without antiphospholipid antibody syndrome (*n* = 39).

b In combination with another second-line treatment (*n* = 1).

c All patients received immune suppressants in combination with other eligible second-line treatments.

## Discussion

In our systematic review of published studies, which included 308 ITP patients at high risk of thrombosis due to immunological mechanisms on at least one eligible second-line treatment, we observed a clinically meaningful frequency of thrombosis (10%, 95% CI 5–19), with no significant differences between the frequencies of patients with arterial and venous thrombosis (7% *vs* 6%, *P* = 0.49).

Our systematic review focused on patients deemed at high thrombotic risk due to immunological mechanisms, such as aPL positivity and coexistence of ITP with immune-mediated diseases which can further increase the risk of thrombosis, such as SLE and APS. A direct comparison of the thrombosis frequency of the examined population with unselected patients with ITP was beyond the scope of this study. Moreover, annualization of thrombosis rates was impossible with available data. Nevertheless, the thrombosis frequencies that we found appear to be higher compared with that of unselected pITP populations [3, 40, 41]. For instance, Ruggeri *et al.* reported a 5-year incidence of venous and arterial thrombosis of 1.4% and 3.2%, respectively, in a cohort of 986 patients with pITP who had received at least one line of therapy [41]. Similarly, Sarpatwari *et al.* found a cumulative incidence of first thrombosis of 6.1% in a cohort of 1070 adults with pITP from 1992 to 2007 with a median follow-up of 48 months [3]. In users of TPO-RAs, the rate of thrombosis has been estimated at 7.5 events per 100 patient-years with romiplostim and 3.17 events per 100 patient-years with eltrombopag [40]. On the other hand, a recent retrospective cohort study found that secondary ITP was independently associated with thrombosis [42]. Moreover, in patients with pITP, lupus anticoagulant positivity has been associated with a pooled odds ratio of thrombosis of 6.11 (95% CI 3.40–10.99) [4].

When stratifying high-risk ITP patients by administered second-line treatment, the cumulative frequency of thrombosis appeared remarkably similar across all treatment groups: 16% (95% CI 11–22) in patients treated with TPO-RAs, 14% (95% CI 3–35) in those treated with rituximab, 11% (95% CI 2–28) in patients on immune suppressants, and 12% (95% CI 4–24) in those who underwent splenectomy. Similar findings were observed when examining SLE patients, who made up most of our cohort (232 out of 308 eligible patients), although point estimates should be interpreted with caution, given the overlapping confidence intervals and the non-negligible use of combination treatments (especially in the case of immune suppressants). Notably, investigated treatments included both treatments that have been associated with the occurrence of thrombosis, such as TPO-RAs and splenectomy, and others that have not, such as rituximab and immune suppressants [15–26]. Taken together, our findings, despite not drawing firm conclusions regarding treatment-related harm or safety, allow to hypothesize that thrombotic risk is primarily driven by the underlying autoimmune disease rather than by specific second-line treatments. In fact, the observed thrombotic rate may reflect the pro-thrombotic immune *milieu* of this at-risk population. However, the contribution of second-line treatments cannot be disentangled given the observational design, confounding by indication, limited power and incomplete data on concomitant antithrombotic therapies. Other possible explanations for our results include (i) a lack of statistical power to detect differences among the examined subgroups, (ii) the baseline heightened thrombotic risk of these patients, which may attenuate the differential contribution of treatments in the onset of thrombotic events, (iii) confounding by indication, as physicians might tend to prescribe agents typically considered safe in terms of thrombotic risk, such as rituximab and immune suppressants, to at-risk patients and (iv) the possible protective effect of antithrombotic therapies, which we could not assess in this study.

With regards to disease subgroups, we have found clinically relevant frequencies of thrombosis in patients with SLE, SLE with positive aPL not meeting criteria for APS, APS and pITP with positive aPL, ranging from 8% (95% CI 4–16) in SLE to 45% (95% CI 28–63) in APS. These findings are consistent with existing literature [31, 43–49]. Previous studies have reported an increased thrombotic risk in patients with ITP secondary to these autoimmune diseases, especially if treated with TPO-RAs, suggesting a potential additive prothrombotic effect in this subset of patients [31, 43]. Patients with SLE have an increased thrombotic risk due to immune dysregulation and vascular autoimmune damage [44–47]. Moreover, despite the limited number of patients resulting in wide confidence intervals, we observed a remarkable thrombosis frequency in ITP patients with aPL positivity without a diagnosis of APS. This observation was consistent in both pITP patients and in patients with ITP secondary to SLE, suggesting a potentially independent role of these auto-antibodies in enhancing the thrombotic risk, as recently described by others [39]. Although the thrombotic risk associated with different aPL patterns is known to be very different [48, 49], we were unable, for the purposes of this analysis, to better stratify the patients based on aPL characteristics such as persistence, isotype (IgG *vs* IgM), titre (medium-high *vs* low or doubtful) and pattern of positivity (double or triple *vs* single). However, considering that aPL positivity is frequently detected in patients with ITP [50, 51], this finding supports the importance of routinely assessing the aPL profile in ITP patients, particularly in those with additional thrombotic risk factors, to better stratify the thrombotic risk and guide therapeutic decisions.

The results of our systematic review must be read in light of its limitations. All eligible studies were observational studies at critical risk of bias due to uncontrolled confounding of treatment selection. There was considerable heterogeneity in the length of follow-up and the annualization of thrombotic events was not possible. Some patients received more than one second-line treatment or combination therapies, leading to potential overlap across treatment categories and thereby limiting the interpretability of treatment-specific analyses and comparisons across treatment groups. Moreover, some disease subgroups (such as APS and RA) were poorly represented and only one study provided data on high-risk pITP, which is the reason why we did not carry out a meta-analysis for this patient population. In light of the limitations listed above, the very low statistical heterogeneity observed in several analyses should be interpreted with caution, as it may reflect the limited number of pooled studies rather than true clinical homogeneity across populations. We retrieved no eligible studies describing ITP secondary to IBD or high-risk ITP patients treated with fostamatinib, which likely results from the paucity of patients with ITP secondary to Crohn’s disease or ulcerative colitis in clinical practice, the relatively recent introduction of fostamatinib, limiting its use in secondary ITP subgroups and the possibility of publication bias. Additionally, patients with primary ITP and aPL positivity may have been missed if these autoantibodies were not specifically searched or reported in the original studies or mentioned in the article indexing information.

Our results, while pooling existing evidence on an under-investigated and clinically relevant topic, also point to unresolved research questions. With the available data we were unable to investigate the potential impact of age, cardiovascular risk factors and antithrombotic treatments (anticoagulants and/or antiplatelet agents), which have been found to play a significant role in unselected patients with ITP [31, 42–47, 50–52], on the occurrence of thrombotic events in this patient population. Moreover, because we restricted our search to studies published from 2010 onwards, we may have incurred in an underrepresentation of treatments used before TPO-RAs became available in clinical practice. It is also worth recalling that none of the evidence-based current ITP guidelines specifically address the management of ITP secondary to systemic rheumatological conditions such as SLE and APS, arising the possibility that the collected evidence may be influenced not only by the paucity of eligible subjects and publication bias, but also by local expertise, background of treating physicians and regulatory access to second-line treatments. Finally, additional immune-mediated diseases associated with ITP, such as Sjögren’s, could be considered at high thrombotic risk but were not included in this systematic review, representing a potential area for future investigation.

## Conclusions

The results of our systematic review, although limited by the low quality of eligible studies and heterogeneity in follow-up duration, show a clinically meaningful frequency of thrombosis in ITP patients at high thrombotic risk due to immunological mechanisms. Events were reported across all examined treatment options, with similar rates, but in the absence of direct comparative analyses and with variable follow-up no conclusions can be drawn on treatment-related differences. It is, however, plausible that such propensity to thrombosis reflects the underlying pro-thrombotic environment posed by aPL positivity and/or by the associated immune-mediated comorbidities of this unique patient population, rather than direct effects of the administered treatments. Future research should focus on the complex interplay of ITP chronicity and refractoriness, underlying autoimmune *milieu*, patients’ age and comorbidities, and mechanisms of action of administered treatments in the onset of thrombosis.

## Supplementary Material

keag461_Supplementary_Data

## Data Availability

The data supporting the results of this article will be shared on reasonable request to the corresponding author.
