# Supplementary material for: The thrombotic burden of high-risk immune thrombocytopenia patients receiving second-line treatments: a systematic review
Source: Rheumatology (Oxford). 2026 Aug 26;65(9):keag461. doi: 10.1093/rheumatology/keag461 (PMC13563668; doi:10.1093/rheumatology/keag461)
Supplement: Supplementary file 1 [file keag461_supplementary_data.zip › rhe-26-0196-File002.docx]

**SUPPLEMENTARY MATERIALS**

Supplementary Data S1

Supplementary Figure S1

Supplementary Table S1

Supplementary Table S2

Supplementary Table S3

Supplementary Table S4

Supplementary Table S5

**Supplementary Data S1. Research string**

Medline and Embase: (((Immune Thrombocytopenic Purpura) OR (Autoimmune Thrombocytopenia) OR (Werlhof Disease) OR (Thrombocytopenia) OR (Thrombocytopenic)) AND ((Systemic Lupus Erythematosus) OR (Antiphospholipid Autoantibodies) OR (Antiphospholipid Syndrome) OR (Evans Syndrome) OR (Rheumatoid arthritis) OR (Inflammatory Bowel Diseases))) AND ((Thrombopoietin receptor agonists) OR (TPOras) OR (Thrombopoietin mimetics) OR (romiplostim) OR (avatrombopag) OR (eltrombopag) OR (hetrombopag) OR (fostamatinib) OR (rituximab) OR (Splenectomy) OR (Azathioprine) OR (Cyclophosphamide) OR (Mycophenolic Acid) OR (Cyclosporine))

**Supplementary Figure S1. Flowchart of study inclusion**

**Identification of studies via databases and registers**

Records identified from:

Databases (n = 5876)

Medline (n = 580)

Embase (n = 5296)

Records removed *before screening*:

Duplicate records removed (n = 468)

Records marked as ineligible by automation tools or removed for other reasons (n = 0)

**Identification**

Records screened

(n = 5408)

Records excluded by a human based on title and/or abstract screening

(n = 5050)

Reports sought for retrieval

(n = 358)

Reports not retrieved

(n = 24)

**Screening**

Reports excluded (n = 321) for the following reasons:

- reports were congress abstracts (n = 88)
- reports did not report primary outcome events (n = 70)
- described patients did not meet eligibility criteria (n = 68)
- description of fewer than 5 patients (n = 43)
- reports did not provide disaggregated data suitable for data analysis (n = 24)
- language other than English (n = 18)
- lack of original data (n = 10)

Reports assessed for eligibility

(n = 334)

Studies included in review

(n = 13)

**Included**

**Supplementary Table S1. Characteristics of the eligible studies**

| **Study** | **Country** | **Type of study** | **Eligible primary ITP patients**  **(n/ total)** | **Eligible secondary ITP patients**  **(n/ total)** | **Females (n/N, %)** | **Age**  **(median, range)** | **Prior therapy lines**  **(median, range)** | **ITP-specific therapies investigated** | **Follow-up duration, months**  **(median, range)** |
| --- | --- | --- | --- | --- | --- | --- | --- | --- | --- |
| Li R et al, 2011 [27] | China | Retrospective | 0/17 | 11/17 | 10/11 (90.9) | 40  (13-50) | 2  (1-3) | Immune suppressants,  splenectomy | 36  (20-240) |
| Chen et al, 2011 [28] | China | Prospective | 0/10 | 10/10 | 10/10  (100) | 32  (20-53) | NA | Rituximab | 8.3 |
| Gudbrand-sdottir et al, 2012 [29] | Denmark | Retrospective | 0/32 | 2/32 | NA | NA | NA | TPO-RAs,   splenectomy | NA |
| Zhou et al, 2013 [30] | China | Retrospective | 0/20 | 20/20 | 18/20  (90) | NA | NA | Splenectomy | 42  (5-114) |
| Guitton et al, 2018 [31] | France | Retrospective | 0/18 | 18/18 | 14/18  (77.8) | 52  (21-70) | 4  (3-5)* | TPO-RAs | 14.7  (4.7-47.5) |
| Frison et al, 2019 [32] | Italy | Retrospective | 14/233 | 2/233 | 14/16 (87.5) | 50 (21-72) | NA | TPO-RAs, Rituximab, immune suppressants | NA |
| Shobha et al, 2020 [33] | India | Prospective | 0/12 | 12/12 | 11/12  (91.7) | 34.5  (30-43)* | 4  (2-5) | TPO-RAs, immune suppressants | 6 |
| González-López et al, 2020 [34] | Spain | Retrospective | 0/145 | 14/145 | NA | NA | NA | TPO-RAs | NA |
| Fattizzo et al, 2022 [35] | Italy, Denmark, UK, Spain | Retrospective | 0/29 | 29/29 | 10/29 (34.5) | 61  (24-88) | 6  (1-8) | TPO-RAs, Rituximab, immune suppressants,  splenectomy | NA |
| Roussotte et al, 2022 [36] | France | Retrospective | 0/90 | 84/90 | NA | NA | NA | TPO-RAs, Rituximab, immune suppressants,  splenectomy | NA |
| Li W et al, 2023 [37] | China | Retrospective | 0/11 | 7/11 | 5/7  (71.4) | 38  (20-59) | 3  (2-4) | TPO-RAs, immune suppressants | NA |
| Jiang et al, 2024 [38] | China, Japan | Retrospective | 0/20 | 5/20 | 5/5  (100) | 52  (21-66) | 3  (2-4) | TPO-RAs, immune suppressants | 24  (2-48) |
| Marques et al, 2025 [39] | France | Retrospective | 0/80 | 80/80 | 59/80  (73.7) | 48  (11-86) | 1  (0-6) | TPO-RAs | NA |

Abbreviations: ITP, immune thrombocytopenia; TPO-RAs, thrombopoietin receptor agonists; NA, not applicable; UK, United Kingdom.

*interquartile range

**Supplementary Table S2. Subgroup analysis by disease group and treatment: primary and secondary outcome events of high-risk primary ITP patients stratified by eligible second-line treatment***

|  | **Patients with thrombotic events** | **Patients with arterial thrombosis** | **Patients with venous thrombosis** |
| --- | --- | --- | --- |
|  | **Crude proportion,**  **n/N (%, 95% CI)** | **Crude proportion,**  **n/N (%, 95% CI)** | **Crude proportion,**  **n/N (%, 95% CI)** |
| **All patients** | 3/14 (21, 5-51) | 0/14 (0, 0-23) | 3/14 (21, 5-51) |
| **Patients stratified by treatment**  **TPO-RAs**  **rituximab°**  **immune suppressants§**  **splenectomy** | 1/5 (20, 1-72)  1/10 (10, 0-45)  2/9 (22, 3-60)  NA | 0/5 (0, 0-52)  0/10 (0, 0-31)  0/9 (0, 0-34)  NA | 1/5 (20, 1-72)  1/10 (10, 0-45)  2/9 (22, 3-60)  NA |

Abbreviations: ITP, immune thrombocytopenia; CI, confidence interval; TPO-RAs, thrombopoietin receptor agonists; NA, not available.

All estimates in this table are crude proportions based on aggregated counts; no random-effects pooling was performed.

Median follow-up range: NA.

*only one study reported data on high-risk primary ITP

°in combination with another second-line treatment (n=7)

§in combination with other eligible second-line treatments (n=8)

The breakdown of prescribed TPO-RAs was unavailable. The total of thrombotic events stratified by eligible second-line therapy exceeds the number of total thrombotic events because of combination therapies

**Supplementary Table S3. Subgroup analysis by disease group and treatment: primary and secondary outcome events of high-risk secondary ITP patients stratified by eligible second-line treatment**

|  | **Patients with thrombotic events** | **Patients with arterial thrombosis** | **Patients with venous thrombosis** |
| --- | --- | --- | --- |
|  | **Crude proportion,**  **n/N (%, 95% CI)** | **Crude proportion,**  **n/N (%, 95% CI)** | **Crude proportion,**  **n/N (%, 95% CI)** |
| **All patients** | 37/294 (12, 8-17) | 20/265 (7, 5-11) | 13/265 (5, 3-8) |
| **Patients stratified by treatment**  **TPO-RAs***  **rituximab°**  **immune suppressants§**  **splenectomy** | 29/183 (16, 11-22)  2/12 (17, 2-48)  1/19 (5, 0-26)  6/51 (12, 4-24) | 16/138 (12, 7-18)  1/12 (8, 0-38)  1/19 (5, 0-26)  3/51 (6, 1-16) | 16/138 (12, 7-18)  1/12 (8, 0-38)  0/19 (0, 0-18)  3/51 (6, 1-16) |

Abbreviations: ITP, immune thrombocytopenia; CI, confidence interval; TPO-RAs, thrombopoietin receptor agonists; NA, not available.

All estimates in this table are crude proportions based on aggregated counts. The meta-analysed frequency of patients with thrombotic events was 9% (95% CI, 5-19), with low heterogeneity (I^2^=0%, *p*=0.69 for heterogeneity). Patients with arterial thrombotic events: 3% (95% CI, 1-15), I^2^=0%, *p*=0.62 for heterogeneity. Patients with venous thrombotic events: 5% (95% CI, 2-9), I^2^=0%, *p*=1 for heterogeneity.

Median follow-up range: 6-42 months.

*all patients received immune suppressants in combination with other eligible second-line treatments

*avatrombopag (n=1), eltrombopag (n=116), romiplostim (n=54)

§in combination with another second-line treatment (n=1)

The breakdown of arterial and venous thrombotic events was unavailable for 5 eligible patients with thrombotic events. Among the 265 patients with available breakdown of arterial vs venous thrombotic events, the type of thrombosis was not specified in 1 patient. The total of thrombotic events stratified by eligible second-line therapy exceeds the number of total thrombotic events because of combination therapies.

**Supplementary Table S4. Subgroup analysis by disease group: thrombotic events in patients with ITP secondary to systemic lupus erythematosus with positive antiphospholipid antibodies and no antiphospholipid antibody syndrome diagnosis across eligible studies***

| **Thrombosis** | | | |
| --- | --- | --- | --- |
| Study | Patients with events/  Total patients | %  (95% CI) | Between-study heterogeneity, I^2^; p |
| Chen, 2011 [28]  Guitton, 2018 [31]  Frison, 2019 [32]  Shobha, 2020 [33]  Marques, 2025 [39] | 1/4  1/5  1/2  0/1  7/27 | 25 (1-80)  20 (1-72)  50 (0-99)  0 (0-98)  26 (11-46) |  |
| **Crude proportion** | 10/39 | NA | NA |
| **Random-effects pooled estimate** | NA | **26 (14-41)** | **0%; 0.96** |
| **Arterial thrombosis** | | | |
| Study | Patients with events/  Total patients | %  (95% CI) | Between-study heterogeneity, I^2^; p |
| Chen, 2011 [28]  Guitton, 2018 [31]  Frison, 2019 [32]  Shobha, 2020 [33]  Marques, 2025 [39] | 0/4  1/5  1/2  0/1  5/27 | 0 (0-60)  20 (1-72)  50 (0-99)  0 (0-98)  18 (6-38) |  |
| **Crude proportion** | 7/39 | NA | NA |
| **Random-effects pooled estimate** | BA | **18 (9-33)** | **0%; 0.91** |
| **Venous thrombosis** | | | |
| Study | Patients with events/  Total patients | %  (95% CI) | Between-study heterogeneity, I^2^; p |
| Chen, 2011 [28]  Guitton, 2018 [31]  Frison, 2019 [32]  Shobha, 2020 [33]  Marques, 2025 [39] | 1/4  0/5  0/2  0/1  4/27 | 25 (1-80)  0 (0-52)  0 (0-84)  0 (0-98)  15 (4-34) |  |
| **Crude proportion** | 5/39 | NA | NA |
| **Random-effects pooled estimate** | NA | **13 (5-27)** | **0%; 1** |

Abbreviations: CI, confidence interval; NA, not applicable.

Crude totals/proportions are reported descriptively and may differ from random-effects pooled estimates.

Median follow-up range: 6-15 months. Patients were treated with TPO-RAs (n=33), rituximab (n=6), and immune suppressants (n=2).

Between-study heterogeneity was assessed using the I² statistic. The p-value refers to heterogeneity (Wald test for the random-effect variance in the random-intercept logistic model).

**Supplementary Table S5. Subgroup analysis by disease group: Thrombotic events in patients with ITP secondary to primary antiphospholipid antibody syndrome (pAPS) and secondary antiphospholipid antibody syndrome (sAPS) across eligible studies**

|  | **pAPS (eligible studies, n=2 [30,31])** | | **sAPS (eligible studies, n=2 [27,30])** | |
| --- | --- | --- | --- | --- |
|  | Patients with events/  Total patients | %  (95% CI) | Patients with events/  Total patients | %  (95% CI) |
| **Thrombosis** | 2/7 | 29 (4-71) | 3/6 | 50 (12-88) |

Abbreviations: pAPS, primary antiphospholipid antibody syndrome; sAPS, secondary antiphospholipid antibody syndrome; CI, confidence interval.

All estimates in this table are crude proportions based on aggregated counts.

The difference in the proportion of pAPS and sAPS patients with thrombosis was not statistically significant (*p*=0.59).
